# Reexamination of the relationships among neurocognition, self-defeatist beliefs, experiential negative symptoms, and social functioning in a sample of patients diagnosed with chronic schizophrenia and schizoaffective disorder

**DOI:** 10.1186/s12888-024-06003-8

**Published:** 2024-08-13

**Authors:** Kun-Hua Lee, Chuan-Hsun Yu

**Affiliations:** 1 Department of Educational Psychology and Counseling, National Tsing Hua University, 521 Nan-Da Road, Hsinchu City, 30014 Taiwan; 2 Department of General Psychiatry, Yuli Hospital, Ministry of Health and Welfare, Hualien County, Taiwan

**Keywords:** Neurocognition, Self-defeatist beliefs, Experiential negative symptoms, Social functioning, Schizophrenia

## Abstract

**Purpose:**

This study proposed and evaluated a theoretical model for exploring the relationships between neurocognition, self-defeatist beliefs, experiential negative symptoms, and social functioning in individuals with chronic schizophrenia.

**Method:**

The study recruited 229 individuals given a diagnosis of schizophrenia and schizoaffective disorders from outpatient clinics and the day ward of a mental health hospital. After informed consent was obtained, the participants underwent assessments using the backward digit span, the digit symbol, and measures of self-defeatist beliefs, experiential negative symptoms, and social functioning. A structural equation model was applied to assess the fitness of the hypothesized model, with indices such as the goodness-of-fit index, comparative fit index, root mean square error of approximation, and standardized root mean square residual being used for model evaluation.

**Results:**

The hypothesized model had an adequate fit. The study findings indicated that neurocognition might indirectly influence self-defeatist beliefs through its effect on experiential negative symptoms. Contrary to expectations, the study did not observe a direct influence of neurocognition, self-defeatist beliefs, or negative symptoms on social functioning. The revised model revealed the role of experiential negative symptoms in mediating the association between neurocognition and social functioning. However, self-defeatist beliefs did not significantly affect social functioning.

**Discussion:**

Before modifying negative thoughts, enhancement of self-awareness ability can help improve negative symptoms and thereby improve the performance of social functions. Future research should develop a hierarchical program of negative symptoms, from cognition rehabilitation to enhancement of self-awareness, and end with modifying maladaptive beliefs.

## Introduction

In a review article [1], reported that over half of individuals experiencing their first episode of psychosis exhibit negative symptoms, with a third demonstrating persistent negative symptoms. These symptoms are associated with diminished subjective quality of life and hindered rehabilitation [2]. Individuals with schizophrenia often face challenges in relationships with relatives and in maintaining daily functions, primarily because of feelings of apathy and a lack of motivation [3]. indicated that negative symptoms can be prevented from progressing to a residual stage, underscoring the importance of understanding such symptoms’ psychopathology and treatment.

According to the European Psychiatric Association, negative symptoms include Affective Flattening or Blunting”, “Alogia”, “Avolition-Apathy”, and “Anhedonia-Asociality” [4]. The consensus conceptualization of negative symptoms involves two factors: expressive (alogia and blunted affect) and experiential (avolition, anhedonia, and asociality) negative symptoms [5, 6]. The prior study reported that experiential negative symptoms more profoundly influence social functioning than expressive negative symptoms do [7].

Researchers have used the positive and negative syndrome scale and social functioning to assess patients on the schizophrenia spectrum and have discovered experiential negative symptoms to significantly affect social functioning, everyday activities, and vocational functioning [8]. By contrast, research has indicated expressive negative symptoms to have a negligible effect on social and vocational functioning [8, 9]. Another study confirmed the substantial influence of experiential negative symptoms on quality of life in a study involving 275 participants with schizophrenia [10]. Further validated the stronger effect of experiential over that of expressive negative symptoms on social functioning [5].

These findings raise the critical question of how experiential negative symptoms influence social functioning. The core symptoms of experiential deficits include asociality, anhedonia, and avolition, which are collectively categorized as avolition–apathy negative symptoms (AA); [1, 7]. Research has indicated that a positive relationship exists between experiential negative symptoms and neurocognitive deficits [11–13]. Neuroimaging research reveals associations between these negative symptoms and a propensity for selecting low-effort options, along with excessive effort-discounting, implicating brain regions such as the insula, hippocampus, amygdala, reward system, and frontal cortex [14].

Patients with schizophrenia often exhibit withdrawal from interpersonal activities and diminished motivation for engaging in enjoyable activities. A case-control study by [15] revealed that individuals with schizophrenia who experienced heightened levels of avolition and anhedonia exhibited less of an inclination towards seeking out rewarding experiences than healthy controls. Furthermore, a study involving 58 patients with schizophrenia examined the relationships between neurocognitive function, avolition–apathy, expressive deficit negative symptoms (DE), and real-life functioning. The study’s results indicated that when AA and DE were considered, the effect of neurocognition on real-life functioning diminished [16]. Thus, neurocognitive function may be a distal factor in social functioning, whereas experiential negative symptoms could be a proximal factor. Additionally, neurocognitive functions include executive function, processing speed, attention, learning, and working memory. In a past study, schizophrenia patients showed poor working memory and processing speed [17, 18]. Regarding measuring working memory, literature indicated the deficit in the visuospatial working memory of schizophrenia could be measured by a digit span test [19]. Besides, another meta-analysis study also found that the digit symbol test is easy to administer and could be sensitive to the deficit in the processing speed of schizophrenia [20]. In line with the reasons above-mentioned and considering that the participants in our study did not have enough patience and attention, digit span and digit symbol were used to assess the neurocognitive function of our participants.

Evidence regarding the effect of experiential negative symptoms on social functioning, however, is not always consistent, for example, A 3-year follow-up study indicated that the influence of these symptoms might fluctuate over time [21]. In addition [22], discovered that some patients with schizophrenia, despite having reduced engagement in social activities, experienced a certain amount of hedonism [8]. identified other factors that could influence the relationship between experiential negative symptoms and social functioning. A review by [7] highlighted the role of dysfunctional beliefs, such as negative expectations about oneself, the future, or the environment, in diminishing the motivation of individuals with schizophrenia to engage in social activities.

One study conducted a 1-year follow-up study investigating the effect of negative expectations on social withdrawal. The study revealed that negative expectations were predictive of poor social functioning, even after neurocognition was controlled for. This finding emphasizes the importance of considering the role of negative thoughts in social functioning [23]. The cognitive–behavioral therapy (CBT) model of negative symptoms for schizophrenia indicates that defeatist performance beliefs may link neurocognition (i.e., memory, attention, and executive function), negative symptoms, and impaired functioning [24]. Beck et al. (2018) reported that when individuals with schizophrenia fail to meet their performance expectations, they are likely to doubt their abilities, which leads to frustration, reduced motivation, and exacerbated negative symptoms [25]. observed that such patterns can impede daily functioning. However, studies have not consistently demonstrated a clear relationship between neurocognition, self-defeatist beliefs, negative symptoms, and social functioning.

For example, a cross-sectional study by [26] provided evidence supporting the existence of an effect of self-defeatist beliefs on negative symptoms, whereas [27] indicated that defeatist beliefs mediated the relationship between neurocognition and negative symptoms. However, these two studies have reported no relationship between defeatist beliefs and daily functioning. Other studies, such as [28, 29], have reported self-defeatist thoughts to have a direct effect on social functioning. They observed that patients with schizophrenia had higher work motivation and retention rates after participating in CBT-based vocational intervention programs focused on reducing self-defeatist beliefs. Additionally, a neuroimaging study by [30] revealed that dysfunctional beliefs may mediate the relationship between negative symptoms and social functioning.

Although studies on the topic have reported inconsistent results, the present study proposes that self-defeatist beliefs mediate the relationship between negative symptoms and social functioning. This hypothesis aligns with the CBT model of negative symptoms, in which maladaptation is considered to be a reaction to the consequences of negative symptoms, which are perpetuated by dysfunctional beliefs [31–33]. This understanding of negative symptoms is supported by a randomized study by [34], which found that changes in dysfunction correlated with improvements in social functioning.

Few studies have investigated the relationships between neurocognition, self-defeatist beliefs, experiential negative symptoms, and social functioning [35]. argued that self-defeatist beliefs mediated the relationship between motivational and reward processing deficits and negative symptoms, particularly apathy and avolition, rather than that between alogia and blunted affect. A randomized clinical trial identified self-defeatist beliefs to play a mediating role in the relationship between experiential negative symptoms and social functioning [36].

**Fig. 1:**
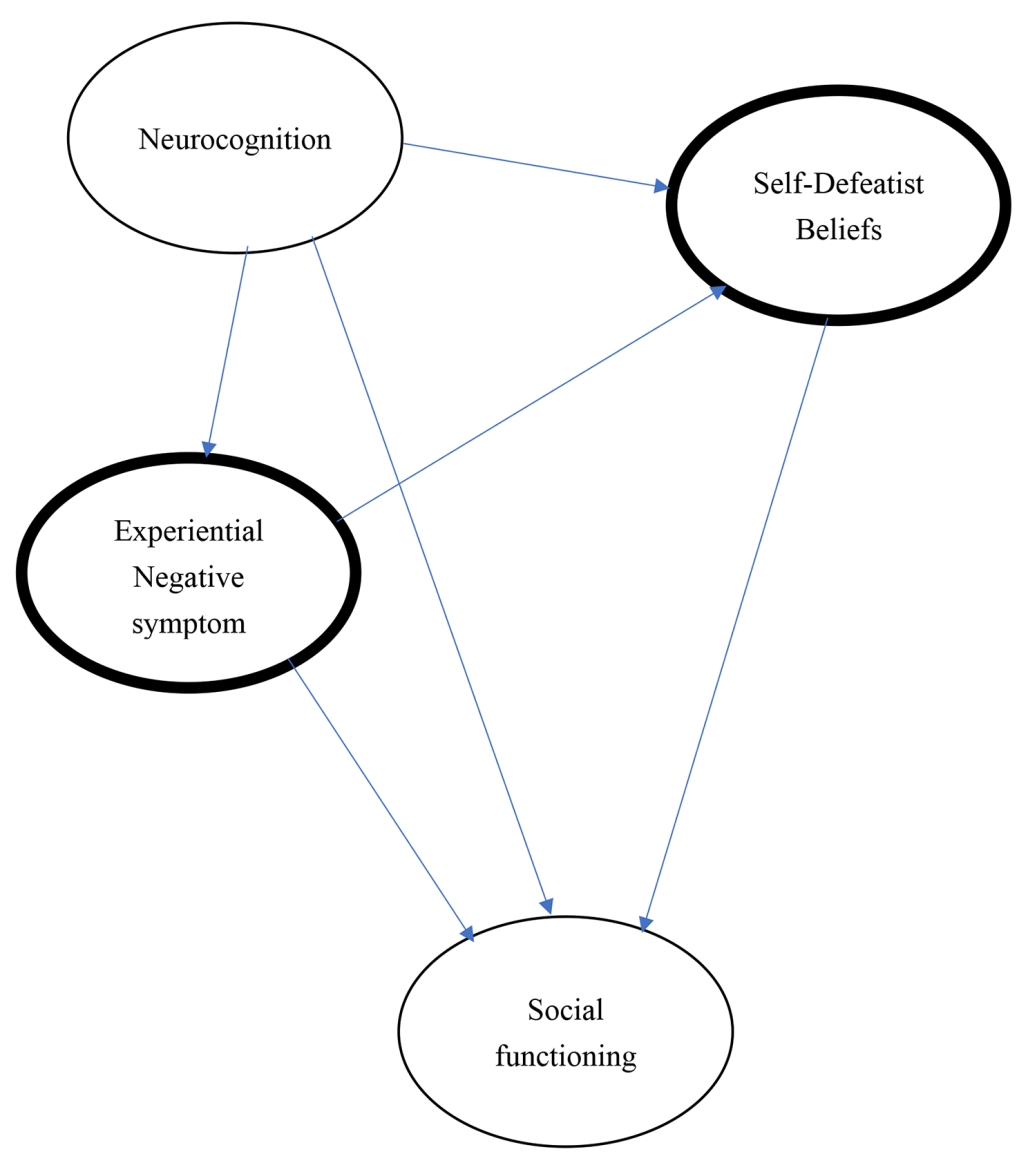
Hypothesized model; Rounded with a thick frame in Experiential negative symptoms and Self-defeatist beliefs: the mediating effects of experiential negative symptoms and self-defeatist beliefs between neurocognition and social functioning

The present study proposed and tested the fitness of a hypothesized model of negative symptoms (Fig. 1) by using structural equation modeling (SEM). It examined the following hypothesized paths: (1) neurocognition may directly affect experiential negative symptoms; (2) neurocognition may directly influence self-defeatist beliefs; (3) experiential negative symptoms may directly influence self-defeatist beliefs; (4) self-defeatist beliefs may directly affect social functioning; (5) neurocognition may influence social functioning; (6) experiential negative symptoms may directly affect social functioning; and (7) self-defeatist beliefs may mediate the relationship between experiential negative symptoms and social functioning.

## Method

### Participants and procedure

According to the Helsinki Declaration of 1975, as revised in 2008, this study was approved by the Institutional Review Board of Yuli Hospital (YLH-IRB-11015) and involved 229 participants with schizophrenia and schizoaffective disorder who were recruited from the clinics and day care ward of a mental health hospital (88 women, 38.4%). All participants provided informed consent. The inclusion criteria were as follows: (1) meeting the *Diagnostic and Statistical Manual of Mental Disorders* (Fifth ed.; American Psychological Association, 2013) criteria for schizophrenia or schizoaffective disorders, (2) being literate, and (3) being able to communicate fluently and coherently. The exclusion criteria were as follows: (1) having delirium or dementia, (2) vividly psychotic symptoms, (3) major depression episode, or (4) acute phase of illness. The average age of the participants was 52.16 years (standard deviation [SD] = 7.09), and 88 female participants were recruited in this study (38.4%).

### Measurements

#### Demographics

The collected data included age, sex, educational attainment, age of onset, and history of chronic diseases.

#### Neurocognition

To run structural equation modeling (SEM), we adopted two neurocognitive tests to be measurement variables, including working memory and processing speech. In this study, the performance of working memory was examined by digit span, and the performance of processing speed was measured by digit symbol test. The digit span and digit symbol tasks were from the Wechsler Adult Intelligence Scale (WAIS) [37]. The backward digit span asks our participants to recall and rehearse the numbers backward, following the assistant’s instructions for speaking the numbers. The backward scores of the digit span task indicated verbal working memory deficit and executive function impairment [12]. The digit symbol task measured processing speed impairments [38]. The digit symbol asks our participants to complete a single sheet of paper that requires a subject to match symbols to numbers according to a key located on the top of the page. The subject copies the symbol into spaces below a row of numbers. The number of correct symbols within 120 s [39]. The research assistant conducted digit span and digit symbol.

#### Self-defeatist beliefs

The dysfunctional attitude scale, comprising 15 items rated on a 7-point Likert scale, assessed self-defeatist beliefs [40]. Its applicability to patients with schizophrenia was validated and had high reliability [41]. To run structure equation modeling (SEM), DAS was analyzed using exploratory factor analysis (EFA), and two measurement variables were found from EFA. The principal component method with varimax rotation was used to test the model fit. Two subscales, the perfect (items 1, 2, 3, 4, 5, 6, 7, 8, 10, and 15) and high standard (items 9, 11, 12, 13, and 14) subscales, were identified as having total initial eigenvalues = 63.35%. Self-defeatist belief was self-reported by the participants. The Cronbach’s α values for the perfect and high standard subscales were 0.93 and 0.88, respectively.

#### Negative symptoms

This study used the scale to assess negative symptoms (SANS), for which items are rated using a 6-point Likert scale. In this study, SANS was measured by the research assistant. The SANS categorizes negative symptoms under five domains: alogia, affective flattening, avolition–apathy, anhedonia–asociality, and attentional impairment [42]. The two-factor model of negative symptoms [9] measured experiential negative symptoms through anhedonia and avolition components. The reliability values for the two subscales for avolition–apathy and anhedonia–asociality were 0.82 and 0.64, respectively. To examine the relationship among experiential negative symptoms, neurocognitive function, self-defeatist beliefs, and social functioning, only the experiential negative symptoms (anhedonia and avolition components) were analyzed in the present study.

#### Daily functioning

The personal and social performance (PSP) scale assessed social functioning. The PSP scale is designed to evaluate social functioning deficits in schizophrenia, with higher scores indicating superior personal or social activity performance [43]. This study’s Cronbach’s α for the PSP scale was 0.82. In this study, SANS was measured by the research assistant.

### Analysis

An SEM was used to assess the fit of the hypothesized model. The analysis included a correlation test to determine the distributions of the demographic data and measured variables and to investigate the relationships between these variables. The model’s goodness-of-fit was evaluated using the goodness-of-fit index (GFI > 0.9), comparative fitness index (CFI > 0.9), root mean square error of approximation (RMSEA < 0.05), and standardized root mean square residual (SRMR < 0.05; [44]. The significance level was set at 0.05. Bootstrap maximum likelihood was applied to investigate mediating effects.

## Results

### Demographic data

Most of the participants in this study were men, and more than half of the patients reported a history of hyperlipidemia (52.4%). Regarding education level, the largest proportion of participants had a junior high school education (42%). The average age of onset was 24.58 years, indicating long-term experience with schizophrenia in the study population (Table 1).

**Table 1:** Distributions of demographic variables and measured variables

Variables	Means/Standard Deviation/ minimum-maximum	Numbers (%)
Age	52.16/7.09/23–60	
Chronic diseases		
Hypertension		71/31.6
Diabetes		71/31
Heart Disease		95/41.5
Gastrointestinal diseases		113/49.3
Hepatitis		45/19.7
Hyperlipidemia		120/52.4
Stroke		0/0
Gender		
Male		141/61.6
Female		88/38.4
Educational level		
None		21/9.4
Elementary school		78/34.8
Junior high school		94/42
Senior high school		25/11.2
College		6/2.7
Master above		0/0
Onset	24.58/8.72/12–58	
Negative symptoms		
Experiential	11.08/4.14/0–22	
Self-defeatist beliefs		
Perfect	3.79/1.54/0.9-7.0	
High standard	3.76/0.81/0.6–5.8	
PSP	14.37/2.97/4–23	
Backward of digit span	4.09/2.28/0–14	
Digit Symbol	34.72/21.53/0–95	

%: Percentages

As indicated in Table 2, the perfect subscale of self-defeatist beliefs was negatively correlated with experiential negative symptoms. Additionally, the digit symbol task and social functioning exhibited significant correlations with the high standard subscale of self-defeatist beliefs. Social functioning was negatively associated with experiential negative symptoms and the digit symbol task.

**Table 2:** Correlation coefficients of measured variables

	Age	Onset	Experiential negative symptoms	Perfect subscale	High standard subscale	Digit span	Digit symbol	PSP
1	-							
2	0.29**	-						
3	0.09	-0.06	-					
4	0.02	0.10	-0.19**	-				
5	-0.04	-0.01	0.09	0.31**	-			
6	-0.08	-0.11	-0.28**	0.16*	0.12	-		
7	-0.13*	-0.06	-0.18**	0.22**	0.15*	0.48**	-	
8	-0.10	-0.12	0.14*	-0.04	-0.14*	-0.12	-0.18**	-

1: age; 2: onset of schizophrenia or schizoaffective disorder; 3: Experiential negative symptom; 4: perfect subscale of self-defeatist beliefs; 5: high standard subscale of self-defeatist beliefs; 6: backward digit span task; 7: digit symbol task; 8: PSP; *: *p* < 0.05; **: *p* < 0.01

### Hypothesized model

**Fig. 2:**
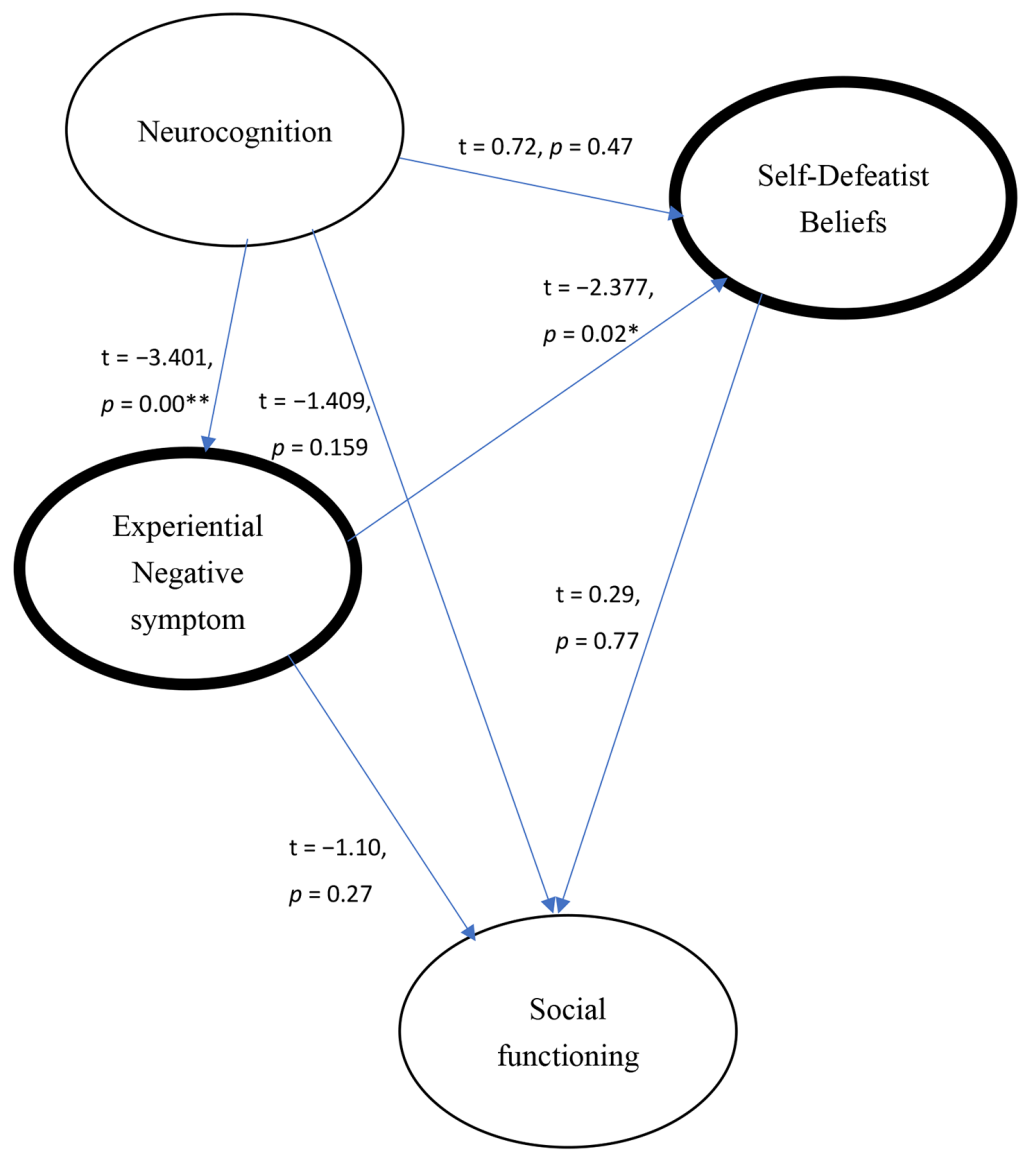
Coefficients of hypothesized model; *: *p* < 0.05; **: *p* < 0.01. Rounded with a thick frame in Experiential negative symptoms and Self-defeatist beliefs: the mediating effects of experiential negative symptoms and self-defeatist beliefs between neurocognition and social functioning

The SEM results indicated adequate model fit, with the following index values: χ^2^ = 28.86, *p* = 0.472; GFI = 0.975 (> 0.90); CFI = 1.000 (> 0.9); RMSEA = 0.000 (< 0.05); SRMR = 0.0397 (< 0.05). Among the hypothesized paths,

1. From neurocognition to experiential negative symptoms (t = − 3.40, *p* < 0.001) and experiential negative symptoms to self-defeatist beliefs (t = − 0.238, *p* = 0.02) were significant.
2. Unexpectedly, several paths did not reach significance (Fig. 2)
  1. neurocognition → self-defeatist beliefs,
  2. neurocognition → social functioning,
  3. experiential negative symptoms → social functioning,
  4. self-defeatist beliefs → social functioning.

### Revised model of negative symptoms

Two nonsignificant paths (neurocognition → self-defeatist beliefs and neurocognition → social functioning) were removed from consideration. For two reasons, we still maintained the link between experiential negative symptoms and social function. The past study supported the effect of negative symptoms on social functioning [5], and the experiential negative symptoms were significantly related to social functioning. We kept the link between experiential negative symptoms and social functioning by examining the relationship between experiential negative symptoms and social functioning.

A revised model was proposed as follows:

1. neurocognition → experiential negative symptoms,
2. experiential negative symptoms → self-defeatist beliefs → social functioning,
3. experiential negative symptoms → social functioning,
4. The revised model had improved fit indices: χ^2^ = 30.89, *p* = 0.47; GFI = 0.97 (> 0.90); CFI = 1.00 (> 0.90); RMSEA = 0.000 (< 0.05); SRMR = 0.048 (< 0.05).
5. All hypothesized paths were significant, except that from self-defeatist beliefs to social functioning (Table 3).

**Table 3:** Coefficients of paths in the revised model

Path	t	*p*
Neurocognition → Experiential negative symptom	−3.28	0.001**
Experiential negative symptoms → Self-defeatist beliefs	−3.04	0.002**
Experiential negative symptoms → Social functioning	2.308	0.021*
Self-defeatist beliefs → Social functioning	0.11	0.92

*: *p* < 0.05; **: *p* < 0.01

## Discussion

This study proposed a hypothesized model to investigate the relationships between self-defeatist beliefs, negative symptoms, neurocognition, and social functioning using an SEM. The proposed model demonstrated a satisfactory GFI, and the revised version supported the effect of experiential negative symptoms on social functioning. However, the results did not confirm a mediating effect of self-defeatist beliefs on the association between experiential negative symptoms and social functioning, which was an unexpected finding.

The current study has several key findings. First, the appropriate GFI in our hypothesized model aligns with the CBT model of negative symptoms, indicating a relationship between experiential negative symptoms and self-defeatist beliefs [23]. However, our results did not reveal a mediating effect of self-defeatist beliefs in the relationship between experiential negative symptoms and social functioning. In other words, the effect of negative symptoms on social functioning could rely more on neurobiological deficits rather than the effects of self-defeatist beliefs. A cross-sectional study investigated the effect of different levels of neurocognition and negative symptoms in chronic schizophrenia. It revealed associations between moderate experiential negative symptoms, impaired cognitive function, and social functioning [45]. Although the CBT model assumes individuals with schizophrenia might struggle more with daily challenges and social interactions, such individuals may have stronger self-defeatist beliefs arising from worsening negative symptoms and impaired neurocognitive functions [46]. Self-defeatist beliefs could occur in different mental illnesses, for example, depression [47, 48] or later-onset psychosis [49]. Thus, self-defeatist beliefs could not be specific to negative symptoms and social functioning. Further study could focus on the effects of neurocognitive deficits between negative symptoms and social functioning.

Second, the revised model highlights the effect of experiential negative symptoms on social functioning, indicating that negative symptoms significantly contribute to neurocognitive deficits [13, 22]. However, the current model did not incorporate social cognition. Literature indicated neurocognition could be overestimated than social cognition, and social cognition could be closer to schizophrenia’s real world and social functioning [50]. In other words, social cognition could reflect the experiences in real life of patients with schizophrenia. Studies, such as [51], have indicated that social awareness may mediate the influence of neurocognition on negative symptoms. Besides, mindfulness-based practice could also improve the severity of negative symptoms and facilitate self-awareness and social awareness in schizophrenia [52]. Therefore, future studies should investigate the relationships among neurocognition, social cognition, belief systems, and negative symptoms.

Third, our findings revealed that neurocognition does not directly influence self-defeatist beliefs, which contradicts the findings of [24]. Theoretically, neurocognitive deficits in individuals with schizophrenia might impede the expected performance of specific behaviors, leading to the development of negative self-schema and subsequent withdrawal and motivation deficits. These withdrawal behaviors are similar to negative symptoms. Therefore, self-defeatist beliefs may stem more from attributions for behaviors associated with negative symptoms than from neurocognitive dysfunction itself. Future experimental or longitudinal studies should investigate the relationship between neurocognitive intervention, self-defeatist beliefs, and negative symptoms.

Fourth, our findings indicate that neurocognition has a direct influence on experiential negative symptoms, a conclusion supported by neuroimaging studies [53]. To our knowledge, research investigating the effect of cognitive rehabilitation on experiential negative symptoms and its integration with changes in self-defeatist beliefs remains limited. Future outcome studies or longitudinal research could explore these relationships.

Fifth, the revised model confirms the relationship between experiential negative symptoms and social functioning; this finding aligns with the two-factor model of negative symptoms [5]. Negative symptoms are related to social functioning and self-defeatist beliefs [25]. Therefore, according to the CBT framework for negative symptoms, therapists can employ two main strategies for assisting individuals with schizophrenia. One involves revising self-defeatist beliefs through reality testing or seeking alternative thoughts. The other involves emotional regulation through mindfulness-based practices. Mindfulness training can enable individuals with schizophrenia to become more aware of and attuned to their feelings and can thereby improve their life experiences [52]. Additional comparative studies assessing the outcomes of mindfulness-based interventions versus cognitive rehabilitation could clarify the distinct effects of neurocognition and belief systems on negative symptoms.

Sixth, our results did not corroborate previous results regarding the effect of self-defeatist beliefs on social functioning [33]. Research has indicated that self-defeatist beliefs can exacerbate negative cognition, influence affect management (e.g., rumination and mindfulness), and lead to a lower likelihood of pursuing goal-oriented behaviors [54]. Future research should therefore investigate the potential moderating role of self-defeatist beliefs on the relationships between negative symptoms, social functioning, and mood status.

In summary, the present study partially confirms the effect of negative symptoms on self-defeatist beliefs and elucidates the relationships between neurocognition, experiential negative symptoms, and social functioning. These findings offer insights into the potential mechanisms underlying the CBT model of negative symptoms, and they may guide the development of different intervention approaches to ameliorate these symptoms. However, several limitations in this study warrant consideration. First, the cross-sectional design prevented us from making causal inferences regarding the relationships between the variables. Longitudinal research could provide more comprehensive insights into such causality. Second, this study did not include biological markers or neuroimaging data; including such information could enrich future studies. Third, the sample size was somewhat limited; subsequent research with a larger sample size could further validate the current findings. Fourth, we did not measure depressive symptoms in our participants, so we could not exclude the confounding effects of depressive symptoms. Fifth, we did not rate the interrater reliability of PSP and SANS. Sixth, there were limited neurocognitive examinations in our study due to the characteristics of our participants (i.e., less patience), so more diverse measurements of neurocognition could be applied in future studies. Despite these limitations, the present study provides valuable directions for future research into the effect of self-defeatist beliefs on negative symptoms in schizophrenia.

## Data Availability

The data sets generated and analyzed during this study are not publicly available because of the privacy considerations of the Institutional Review Board. However, they can be obtained from the corresponding author upon reasonable request.
